# Comparison of a sports-hydration drink containing high amylose starch with usual hydration practice in Australian rules footballers during intense summer training

**DOI:** 10.1186/s12970-018-0253-8

**Published:** 2018-09-21

**Authors:** Sinead Mary O’Connell, Richard John Woodman, Ian Lewis Brown, David Julian Vincent, Henry Joseph Binder, Balakrishnan Siddartha Ramakrishna, Graeme Paul Young

**Affiliations:** 1 Flinders University, GPO Box 2100, 5001 Adelaide, Australia; 2 Flinders Centre for Epidemiology and Biostatistics, Flinders University, GPO Box 2100, 5001 Adelaide, Australia; 3 Flinders Centre for Innovation in Cancer, Flinders University, Adelaide, SA Australia; 4 Department of Internal Medicine, Yale School of Medicine, P.O. Box 208019, New Haven, CT 06520 USA; 5 Institute of Gastroenterology, SRM Institutes for Medical Sciences, Vadapalani, Chennai, 600 026 India

**Keywords:** Hydration, Resistant starch, Sports drink, Footballers

## Abstract

**Background:**

Fluid deficits exceeding 1.6% can lead to physical and cognitive impairment in athletes. Sport drinks used by athletes are often hyper-osmolar but this is known to be suboptimal for rehydration in medical settings and does not utilize colonic absorptive capacity. Colonic absorption can be enhanced by fermentative production of short chain fatty acids (SCFA) from substrates such as high amylose maize starch (HAMS). This study therefore compared, in elite Australian Football League (AFL) players at the height of outdoor summer training, a novel dual-action sports oral rehydration strategy that contained HAMS as well as glucose, to their usual rehydration practices (Control). The primary outcome markers of hydration were hematocrit and body weight.

**Methods:**

A randomized single-blind crossover study was undertaken in thirty-one AFL players; twenty-seven completed the study which was conducted on four days (two days in the Intervention arm and two in Control arm). The Intervention arm was comprised a 50-100 g evening preload of an acetylated HAMS (Ingredion Pty Ltd) followed by consumption of a specially formulated sports oral rehydration solution (SpORS) drink during intense training and recovery. Players followed their usual hydration routine in the Control arm. Quantitative assessments of body weight, hematocrit and urine specific gravity were made at three time-points on each day of training: pre-training, post-training (90 min), and at end of recovery (30–60 min later). GPS tracking monitored player exertion.

**Results:**

Across the three time-points, hematocrit was significantly lower and body weight significantly higher in Intervention compared to Control arms (*p* < 0.02 and *p* = 0.001 respectively, mixed effects model). Weights were significantly heavier at all three assessment points for Intervention compared to Control arms (Δ = 0.30 ± 0.13, *p* = 0.02 pre-training; Δ = 0.43 ± 0.14, *p* = 0.002 post training; and Δ = 0.68 ± 0.14, *p* < 0.001 for recovery). Between the pre-training and end-of-recovery assessments, the Control arm lost 0.80 kg overall compared with 0.12 kg in the Intervention arm, an 85% lower reduction of bodyweight across the assessment period.

**Conclusion:**

The combination of the significantly lower hematocrit and increased body weight in the Intervention arm represents better hydration not only at the end of training as well as following a recovery period but also at its commencement. The magnitude of the benefit seems sufficient to have an impact on performance and further studies to test this possibility are now indicated.

**Trial registration:**

Trial is listed on the Australian New Zealand Clinical Trials Registry (ACTRN 12613001373763).

## Background

Intense exercise can lead to a loss of 1-3 L of fluid/h and may be further aggravated in warm climates [1]. In Australian Rules football, which typically involves up to 2 h of intense physical activity, players lose between 1.2–3.5% of their body weight in a typical match [2]. A fluid deficit of as little as 1.6% of body weight can lead to thermal stress, impaired cognition, cardiovascular and exercise function, as well as accelerated fatigue and prolonged recovery time [3–7]. For example, the impact of a fluid deficit of 1.6% bodyweight led to a 1.31 min slower running time over 5000 m (*p* = < 0.05) which equated to a 6.7% slower running time compared with the hydrated state **[**5].

Current sports drinks consumed by athletes during and after prolonged physical activity, often have a glucose content much higher than that demonstrated to be the most effective for rehydration in medical settings.

Studies in human volunteers have shown that water absorption from the small bowel is impaired when luminal glucose concentration is higher than 80 mmol/L and may be reversed, changing to active secretion, when osmolality exceeds 250 mmol/L [8]. Furthermore, clinical trials of oral rehydration solutions (ORS) clearly show that hypo-osmolar solutions with no more than 13.3 g/L glucose (compared with ~ 60 g/L of sugars in Gatorade, see Table 1) achieve faster and more effective rehydration in children and adults with acute diarrhea than either water alone or ORS with higher glucose concentrations [9]. As a consequence, hypo-osmolar solutions (see Table 1) now represent the WHO/UNICEF standard for rehydration in medical settings [10]. These hypotonic medically-proven formulations depend on active glucose absorption against a concentration gradient to drive small intestinal absorption of water to correct the water deficit [11]. Many sports drinks contain 30-80 g/L of simple sugar such as glucose, sucrose or fructose (see Table 1 for a typical formulation). The higher composition of simple sugars in sports drinks might provide energy but their high sugar composition seems likely to be suboptimal for hydration and performance enhancement, given the clear evidence from medical settings that hypo-osmolar solutions are better for hydration.

**Table 1:** SpORS Formulation and comparison to formulations for WHO ORS and a selected sports drink; components shown making up to 1 L in drinking water

Item	Hypo-osmolar-ORS (WHO)#	Gatorade	SpORS
NaCl	2.6 g/l^a^	1.14 g/l	1.45 g/l
KCl	1.5 g/l^a^	0.19 g/l	0.4 g/l (as KCl)
TriSodium citrate dihydrate	2.9 g/l^a^	As pot and sod citrate	1.6 g/l
sugar	13.5 g/l glucose	60 g/L	5 g/l glucose
Starch			45 g/L acetylated HAMS^b^
Osmolality	245 mOsm/L^a^	330 mOsm/L^c^	63.7 mOsm/L^d^

^a^UNICEF/WHO 2009

^b^Acetylated HAMS is Hylon VII acetylated 2.5% (Ingredion Pty Ltd), as used in the food industry and accorded GRAS food safety status. This provides a fermentable starch as well as delivering acetate to the colon as resident bacteria split the acetate from the starch backbone

^c^Mettler S, Rusch C, Colombani PC. Osmolality and pH of sport and other drinks available in Switzerland. Schweiz Z Sportmed. 2006;54:92–95

^d^Estimated

The large intestine is capable of absorbing upwards of 5 L per day of water but glucose plays no role in driving absorption in the large intestine. Water and electrolyte absorption in the large intestine requires the presence of short chain fatty acids (SCFAs) which are produced by fermentation of carbohydrates by resident colonic bacteria in the large intestine [12]. Feeding SCFAs is not an option as they are not all palatable and are absorbed by the small intestine before they reach the large intestine. However, it has been shown that ingestion of a starch resistant to digestion (“resistant starch”, RS) such as high amylose maize starch (HAMS), generates substantial amounts of SCFAs in the large intestine [13]. Furthermore, oral rehydration solutions that utilize the absorptive capacity of *both* the large and small intestine (so-called dual-action ORS), by incorporating RS, have been developed and proven effective in the treatment of severe diarrhea in adults and children [9, 12, 14, 15].

This dual-action principle, whereby glucose drives small intestinal fluid absorption and RS-generated SCFA drives large intestinal fluid absorption, provides a new option for improving hydration in athletes. While the mechanism and nature of dehydration and electrolyte losses differ in patients with acute diarrhea compared to that from sweat and transpiration, the use of RS in an appropriate dual-action formulation has the potential to improve hydration during and after strenuous exercise. However, RS is insoluble and could take 6 h to reach the colon. Thus, preloading athletes by consuming RS was undertaken to ensure the ready availability of RS for fermentation at the time that exertion is undertaken.

Therefore, the present study compared a two-part hydration strategy intervention to usual hydration practices (Control) in elite Australian Football League (AFL) players at the height of their outdoor, summer training. The Intervention arm consisted of a RS preload which was consumed the evening before training and a dual-action sports oral rehydration solution (SpORS) that contained an acetylated HAMS, the source of RS, as well as glucose. The rationale behind RS preload consumption the evening before training was that an individual cannot be overhydrated, as they will pass excess fluid through urination but the RS can be preloaded into the large intestine so that when fluids are ingested during intensive exercise, optimal fluid and salt absorption will occur rapidly. A randomized single-blind crossover design was undertaken comparing Intervention with Control arms prior to, at the end of training and after a recovery period. The primary outcome markers of hydration were body weight and hematocrit.

## Methods

### Subjects

AFL club-listed players from the Adelaide Football Club, South Australia, who had entered intense pre-season training were studied in the peak of summer (mid to late January 2014). Temperatures reached at least 34.2 degrees Celsius by 10.20 am on each of the four days of study. On one day the temperature had reached 42.7 °C by 11.30 am.

Inclusion criteria for the study were: formal listing on the players’ roster, scheduled to undertake the full exercise schedule on each day, considered (by the player) to be able to tolerate the anticipated fluid intake, no current gastrointestinal disorder including vomiting and able to consent to participate. Exclusion criteria were: presence of an injury that in any way meant they were not able to complete the training/exercise schedule, receiving diuretics or other drugs considered likely to affect hydration and unwilling/unable to sign the study consent form.

All participants consented to the study which was approved by the Southern Adelaide Clinical Human Research Ethics committee and listed on the Australian New Zealand Clinical Trials Registry (ACTRN 12613001373763). Each participant received a simple language statement prior to the study that included:

1. A statement on the voluntary nature of the study,
2. Background on the rationale of the study and the anticipated outcomes, and
3. A description of the measurements that would be taken and an explanation of them.

### Study design

A single-blinded cross-over study was performed over four separate days; two Control days and two Intervention days. During the Control days the players followed their usual hydration practices consuming their preferred hydration fluids (water, Gatorade or a combination of both). During the Intervention days the players consumed the RS preload the evening before and the SpORS drink on the day of training. Players were not blinded during the study, due to the nature of the RS but all those taking measurements were.

Players were randomly assigned to the order in which they undertook the Control or Intervention periods using a simple computer generated randomization table which generated a list of 1 's and 0 's. This table was merged with an alphabetical list of participants randomly assigning them to either the Intervention or Control arm. Each study period consisted of two separate days within the same week. There was a one week washout between Control and Intervention. The first period took place in the second week of January, and the second period in the fourth week of January. A washout period has been considered best practice in trials involving fermentable starches to allow for clearance of the starches from the large intestine by the time of the control period [16–18].

### Intervention

The intervention consisted of two components: a RS preload the night before and a SpORS drink for the day of training. The RS *preload* involved consumption of an HAMS-containing flavored milk containing a commercially available source of resistant starch (acetylated Hylon VII from Ingredion Pty Ltd. (Melbourne Australia), Cat. No. 06460B06CE see footnote to Table 1) the night before the training session. Players were instructed to mix the HAMS (100 g preweighed and provided in a ziplock bag) into commercially available flavored milk 600 ml. The flavored milk acted to mask the mild flavor of the RS to ensure palatability. As this was a pragmatic study of effectiveness rather than efficacy, players were allowed to self adjust the actual amount consumed between 300 and 600 ml (50 to 100 g of HAMS). Four players experienced abdominal discomfort (including flatus) when consuming a 100 g RS preload. For this reason, all players were then given the option of consuming 50 g or 100 g.

The SpORS *drink* (formulated as shown in Table 1 with 5 g/L glucose and 45 g/L RS) was consumed from half way through each training session and during the recovery period until final measurements were taken. Players were instructed to consume as much as tolerable. Use of high glucose gels and drinks were suspended from halfway through the training session in the Intervention Arm but water was allowed throughout.

### Study procedures

Study nurses were available in the training facility to monitor participants and take measurements on each of the study days. Nurses followed participants carefully so as to standardize the timing and order of measurements as much as feasible without unduly interfering with the training schedule.

### Training

Training programs comprising intense endurance work was prescribed for each player on 3 set days in each week which were not modified because of this study. Players were studied on two of these three days in the study week when intense training was scheduled. They did not alter their nutritional programs.

On each day, the training consisted of a briefing, a warm-up, an on-field 80–120 min training session (with breaks of 2–3 min every 20 min) involving sprinting, skills development and actual play, all followed by a recovery phase of 60 min. While these varied to some degree between players based on their specific roles in the team, they were generally consistent for each player.

The amount of physical exercise undertaken was monitored using an electronic GPS tracking system (Catapult Sports) on each player that included measures of distance travelled, total duration of training time, sum of high intensity (> 17 km/h) distance travelled and sum of very high intensity (> 23 km/h) distance travelled.

Adherence to intervention and subjective measures were assessed using a structured questionnaire administered by a study nurse at the time of each study day’s final measurements. This addressed the following areas: five subjective questions using a visual analog scale addressing thirst, bloating, feeling of refreshment, stomach upset and tiredness (kindly provided by Kalman et al. [19]).

### Timing of measures

Measurements were taken each morning after arrival at the training facility in all participants (within 15 min prior to the commencement of training) (the “pretraining” assessment). Measurements were repeated within 15 min at the end of training (“post-training” assessment) and at the end of recovery, ~ 30–60 min after the end-of-training (“recovery” assessment).

### Measurement details

Urine specific gravity was measured immediately at each of the three Assessments. A urinometer was used to measure specific gravity in a temperature-controlled room once all collections were available. Body weight was measured at the same times using a single electronic scale with digital readout, wearing shorts only, immediately after urination. Hematocrit was measured using finger-prick collection of blood into a capillary tube immediately after weighing. Tubes were spun at 13,000 rpm for two minutes in a capillary centrifuge and the percentage of blood volume occupied by erythrocytes determined using a graduated reading device.

### Withdrawal criteria

Subjects were allowed to withdraw at any time during the study, freely & without prejudice, if they developed intolerance to the products supplied, if they experienced acute onset of any disease, or due to injury.

### Statistical analysis

Analysis on an intention-to-treat basis was performed using Stata (StataCorp, Texas, USA, version14.0). A mixed effects model was used to assess differences in each outcome between Control and Intervention arms at each Assessment time point. Fixed effects terms in the model included time as a categorical variable (assessment times being made on the training day: pretraining, post-training or after recovery), treatment arm, order of treatment (1 or 2) and exercise time (as a continuous variable). An interaction term between treatment arm and time was included to assess the effects of treatment at the end of each period. Subject ID was included as a random intercept term to account for the within-subject correlation. In a sensitivity analysis, adjustment for measures of distance covered and average heart rate were also included in addition to exercise time, in order to control for possible differences in the amount and intensity of training performed under the two conditions. Results of these analysis were however substantively similar, indicating that these factors were not significantly associated with the received treatments, and therefore only the results of the primary analysis are presented. Descriptive statistics are presented as estimated mean difference (Δ) ± SD. A two-sided type 1 error rate of *p* < 0.05 was considered statistically significant.

Differences between groups for the survey questions on subjective measures were assessed using a mixed effects model with study arm and period (first 2 days versus second 2 days) as fixed effects and the Subject ID as a random effect. The mean differences between Intervention and Control arms were assessed using the predicted marginal means between the two groups. Testing for an arm times period interaction effect was also performed and included this in the final model when significant. The fit for each model was assessed by both the residuals and random effects for normality.

### Sample size

The study was powered to detect a meaningful difference in bodyweight between treatments at either the end of the training session or following the warm-down, of one kilogram. Given a SD of differences in bodyweight of 2.0, a sample size of thirty four would be required to provide 80% power using a paired t-test with a two-sided Type 1 error rate of alpha = 0.05. A sample size of thirty four would also provide 80% power to detect a difference in means for USG of 0.01 assuming a standard deviation of differences of 0.02, using a paired t-test with a two-sided Type 1 error rate of alpha = 0.05. Power was slightly higher than this given that four measures rather than only two were performed on each subject.

## Results

### Subject disposition

Thirty one players consented to participate in the study. Two withdrew prior to the start of the study. One withdrew due to a sports injury unrelated to the study intervention and one for rehabilitation which precluded him from intense exercise for prolonged periods. Twenty-seven players who participated on each of two days during Control and Intervention arms regardless of how much preload was consumed were included in the final analysis.

The mean (±SD) age of player was 22.85 ± 3.36 years. The mean (±SD) weight at the commencement of training in the Control arm was 87.22 ± 8.68 kg.

### Hematocrit

**Fig. 1:**
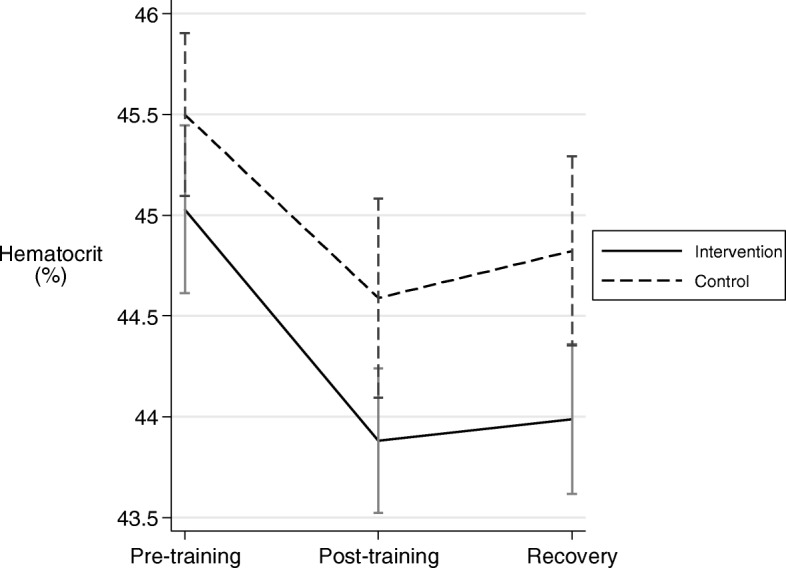
Mean hematocrit for the Intervention and Control arms at assessment points immediately pre-training, at the end of training and at the end of recovery. Intervention vs Control: *p* = 0.02 overall, *p* = 0.34 at pre-training, *p* = 0.14 at post-training and *p* = 0.08 at conclusion of recovery. Vertical lines are standard errors. *N* = 50, 45, 45 for Control, and N = 50, 46, 43 for Intervention for each time-point respectively

Mean hematocrit is shown for Intervention and Control arms in Fig. 1. Hematocrit was significantly lower for Intervention versus Control arms overall across the three time points (Δ = − 0.77 ± 0.32, *p* = 0.02). Hematocrit was non-significantly lower at each of the three individual assessment points for Intervention compared to Control arms (Δ = − 0.52 ± 0.54; *p* = 0.34 for pre-training, Δ = − 0.82 ± 0.14; *p* = 0.14 for post training and Δ = − 0.99 ± 0.56; *p* = 0.08 for recovery; mixed effects model comparing arms at each time point) although the difference was almost significant at the final measurement.

The lower overall hematocrit with treatment reflects a higher plasma volume when participating in the Intervention.

### Body weight

**Fig. 2:**
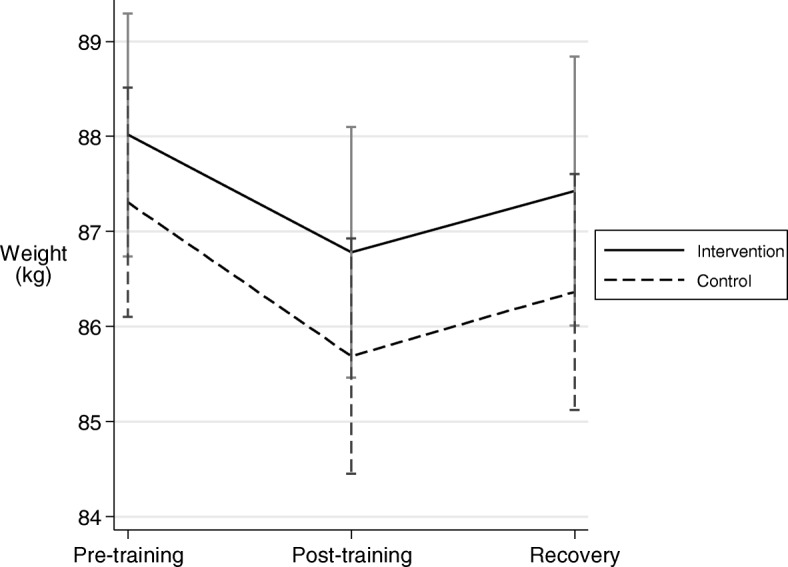
Mean body weights (observed) for the Intervention and Control arms at assessment points immediately pre-training, at the end of training and at the end of recovery. Intervention vs Control: *p* = 0.02 at pre-training, *p* = 0.002 at post-training and *p* < 0.001 at conclusion of recovery. Vertical lines are standard errors. *N* = 50, 45, 45 for Control, and *N* = 50, 46, 43 for Intervention for each time-point respectively

The mean observed body weights for the Intervention and Control arms at each Assessment point of the study are shown in Fig. 2. Weights were significantly heavier at all three assessment points for Intervention compared to Control arms: (Δ = 0.30 ± 0.13; *p* = 0.02 for pre-training, Δ = 0.43 ± 0.14; *p* = 0.002 for post training and Δ = 0.68 ± 0.14; *p* < 0.001 for recovery; mixed effects model comparing arms at each time point). The overall difference for treatment versus control over the three time points was significant (*p* < 0.001).

The treatment effects of the Intervention on weight, together with the higher plasma volume are consistent with better hydration before training, followed by significant benefit at the conclusion of training and after recovery.

Differences in weight, expressed as a percentage of *adjusted* mean body weight are summarized in Table 2. Players in the Intervention arm retained an additional 0.34% to 0.76% depending on the time of measurement. Between the pre-training assessment and the recovery assessment the Control arm lost 0.80 kg overall compared with 0.42 kg in the Intervention arm, a 47% lower reduction across the entire assessment period.

**Table 2:** Adjusted body weights at each Assessment time in each arm, showing percentage changes and significance (mixed effects model)

	Pre-training Assessment (A)		Post-training Assessment (B)		Recovery Assessment (C)	
	Control	Intervention	Control	Intervention	Control	Intervention
Weight (kg) Mean	88.59	88.89	86.95	87.39	87.79	88.47
Weight difference(%), *p*-value	0.30 kg (0.34%), *p* = 0.023		0.43 kg (0.49%), *p* = 0.002		0.68 kg (0.76%), *p* < 0.001	

**Fig. 3:**
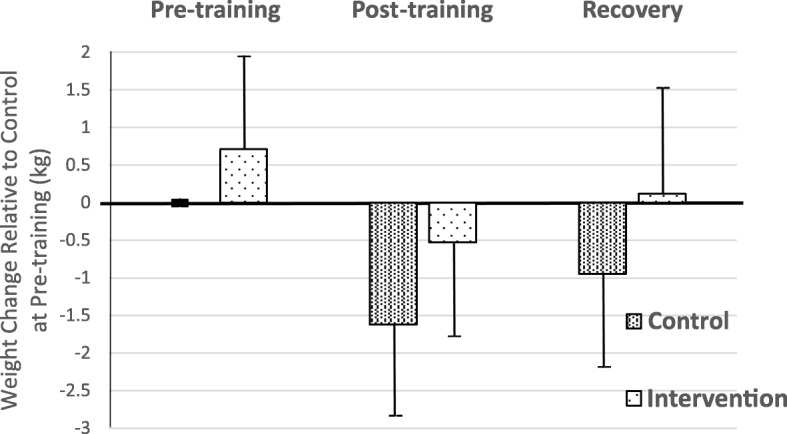
Weight (observed) changes for each Arm (Control and Intervention) at each assessment point, relative to the pre-training measurement in the Control arm. Vertical bars are standard errors

Study design did not allow for a baseline measurement prior to the RS preload consumed on the night before and the relevance of this to the next day of exercise was considered uncertain. However, the body weight in the Control arm at the time of the pretraining assessment can be considered as a baseline for estimating weight changes at other measurement times. It can be seen from Fig. 3, which shows changes in observed body weights for each arm (Control and Intervention) at each assessment point relative to the pre-training measurement in the Control arm, that players in the Intervention arm started out heavier than when in the Control arm and returned to baseline after recovery, whereas when in the Control arm they did not.

### Exercise time and intensity

There were no significant differences in exercise time between the Control and Intervention arms across study days (see Table 3). Analysis of exercise intensity, measured as time spent exercising at greater than 17kph and greater than 23kph, did not find any significant differences between the Control and Intervention arms (Table 3). This data indicates that the exercise time and intensity were not substantially different between treatment groups.

**Table 3:** Mean exercise time and intensity in the Control and Intervention arms as measured across four days for each arm (mixed effects model)

	Control Mean ± SD	Intervention Mean ± SD	*P*-value
Exercise time (mins)	83.1 ± 30.3	93.8 ± 28.5	0.069
Time > 17kph (secs)	1555.2 ± 976.6	1487.3 ± 965.0	0.735
Time > 23 kph (secs)	285.2 ± 181.1	289.0 ± 209.8	0.926

### Urine specific gravity

There were no significant differences in urine specific gravity between the groups at any of the three time points: Δ = 0.0092 ± 0.0102; *p* = 0.36 pre training, Δ = 0.0121 ± 0.0104; *p* = 0.24 post-training, Δ = 0.0182 ± 0.0106; *p* = 0.09 recovery. However, there was a significant difference across all three time points (Δ = 0.0132 ± 0.0062; *p* = 0.03) (data not shown).

### Subjective data

Table 4 shows the mean responses for the subjective Likert scale data during both the Control and Intervention arms, as well as the estimated effect for Intervention versus Control. All models for the questions related to subjective feelings showed good fit based on the level 1 and level 2 residuals. There were no significant differences between treatments in the mean response for Question 1 (Level of thirst) (*p* = 0.21), Question 3 (Feeling of being refreshed) (*p* = 0.83) and Question 5 (Level of fatigue) (*p* = 0.66). The level of bloated-ness was significantly higher in the Intervention compared to the Control arm (*p* < 0.001) as was abdominal discomfort (*p* < 0.001).

**Table 4:** Mean ± SD from Likert scale data (1 = Not at all to 5 = extremely) on subjective feelings (*n* = 27)

	Control Mean ± SD	Intervention Mean ± SD	Estimated Δ^1^ Mean ± SE	*P*-value for Δ^1^
1: Level of Thirst	2.9 ± 0.7	2.7 ± 0.7	−0.20 ± 0.16	0.21
2: Level of Bloating	2.0 ± 1.1	2.8 ± 1.2	0.83 ± 0.24	< 0.001
3: Sense of Refreshment	2.5 ± 0.9	2.5 ± 0.8	0.04 ± 0.16	0.83
4: Stomach discomfort	1.6 ± 0.8	2.5 ± 1.0	0.95 ± 0.17	< 0.001
5: Sense of fatigue	3.2 ± 1.0	3.1 ± 1.0	−0.08 ± 0.19	0.66

^1^The difference, Δ, for Intervention versus Control was obtained using a mixed effects model with arm and period as the fixed effects and subject as a random effect (see Methods)

## Discussion

In elite high-performance AFL footballers undertaking intensive pre-season training at the peak of an Australian summer, dual-action SpORS hydration drink with an RS preload on the evening before resulted in body weights that were significantly heavier at all three assessment points (pre-training, post training and post recovery) and a consistently lower hematocrit, compared with the Control period when following their usual hydration practices. These results reflect better hydration before training and at the completion of training as well as 30–60 min after commencement of the post-training recovery period.

ORSs containing HAMS have been established to improve outcomes in severe diarrhea in adults and children where the mechanism is high fluid and electrolyte loss even in the face of fever and vomiting, and where dehydration is often present before hydration is commenced [12, 14, 15]. This study is the first to demonstrate the benefit of including HAMS in the sports setting. The mechanisms of fluid and electrolyte losses relate to sweating and transpiration in the sports context and the nature of the electrolyte loss is different from that in the medical setting, hence the adjustment in the electrolyte composition to match that of existing sports drinks. Despite this difference in the pathogenesis of fluid losses, and the pragmatic nature of this effectiveness study, the SpORS Intervention was successful. In the sports setting, dehydration due to vomiting or pathophysiological disturbances causing diarrhea are unlikely; thus it is possible to intervene prior to commencing dehydrating physical exertion both to optimize hydration at commencement as well as to prevent dehydration during exertion.

There are many types of resistant starch [20]. Acetylated HAMS (HAMSA) was selected for this study because it is readily fermented to SCFA by the colonic microflora [13, 21], and the presence of the additional acetate covalently bonded to the HAMS provided an added immediate boost to the rehydration once the HAMSA reaches the colon. It was also chosen because of its proven medical benefits, tolerability in humans and a long history of safe use in foods and its corresponding approved regulatory status as GRAS (generally regarded as safe) by the US Food and Drug Administration. No other RS meets these criteria.

Our primary markers of hydration, namely body weight and hematocrit, were significantly better in the Intervention than the Control periods at the pretraining assessment, consistent with better hydration due to the ingestion of RS the evening before. The presumed mechanism is active fermentation in the colon overnight with increased SCFA production, as previously demonstrated in healthy subjects [16], resulting in better fluid absorption in the colon. This also ensures presence of RS in the colon at the time of exertion and consumption of water (in any form).

The benefit of SpORS on body weight and hematocrit relative to control was maintained during exercise and recovery. Players were instructed to drink as much as they could during this period and given the circumstances of the training, their actual fluid consumption was not able to be determined with precision. During the Control arm, they followed their own usual personal practice which was highly variable in volume, mostly comprising water or Gatorade but also comprised of other personally preferred drinks. It should be noted though that they were asked not to consume these during the second half of exercise or during recovery in the Intervention periods and instead only consume SpORS and water.

While it was not possible to measure total body water content, total body weight is an indirect measure of this. The improvement in hematocrit, the most direct measure of hydration used here, was apparent at all times assessed. Importantly, this was true at the end of the recovery period at which time, blood flow would have been rediverted away from muscles and back to visceral organs, a shift that seems likely to enhance fluid absorption from the gut.

Subjective measures did not reveal any benefits for thirst, refreshment or fatigue, which might be expected under such circumstances but bloating and discomfort were scored higher in the Intervention period. This is an anticipated effect of RS [13, 20, 22] and indicates good compliance with the SpORS Intervention. Players did not find this to be unacceptable, except several who could not tolerate the full 100 g preload. A number of studies reporting these symptoms in subjects consuming RS indicate that after a short period of regular consumption, these symptoms settle [22]. Further studies with repeated use, exploring tolerability of different preload doses and different timing of those doses, are now indicated to determine if symptoms do settle and if there are alternatives to consumption the night before. The decision to ask players to consume it the night before was based on the known oral-cecal transit time of around 6–8 h for solid foods.

The strengths of this study centered around the crossover design, the two intervention and control periods, the short timeframe of the study and its execution in extremely hot weather. No period effects that indicated the effect of treatment were different for the first or second period were observed.

The main weakness was that this was a single blinded study with players being aware of the study arm they were in on each day; it is possible that this could have influenced the amount of fluid they decided to consume. However, the measures reported were objective and not subject to recording bias as the recorders were blinded to the study arm which applied to each participant. The exact intensity of the training on each day was unable to be regulated, however our measures of exercise time and intensity indicated that there were no major differences for the two study conditions (see Table 3). Furthermore, a sensitivity analysis adjusting for intensity of effort made no difference to the results (see Methods).

In the context of undertaking the study in the real-world environment of training, it was not possible to rigidly apply time periods that corresponded to the precise minute, to ensure that exertion was identical between participants. Similarly, it could not determine exactly how much fluid and/or RS had been consumed. However, this reflects the “real” context of training and competition and differences were demonstrated despite this. These constraints also meant that analyses were limited to the measures undertaken in the study and more sophisticated measures of body water content were not practical. Urine collection was problematic in this study and urine specific gravity has been shown to be unreliable over short periods as applied here and does not give an accurate perspective at a given point in time, whereas hematocrit does. [23]. While there was a significant difference across the three timepoints, the actual difference was very small and likely to be of no physiological significance. Based on our observations, undertaking further studies of the SpORS strategy in highly controlled exercise environments with standardized workloads, tightly controlled timing of measurement and efforts to assess fatigue and consequences for performance are now indicated.

The physiological changes associated with dehydration affect player performance [4–7, 24, 25]. The level of hydration when commencing exercise is also important as relative rate of oxygen uptake, heart rate and rate of perceived exertion were increased if dehydrated when commencing exercise [26]. Dehydration also leads to 13% slower time-trial performance and accelerated muscle glycogen use in cyclists [6], impaired cognitive ability [27], degraded aerobic performance, high-intensity endurance and sports-specific skills [28]. Recovery kinetics of physical performance is also compromised [29].

It is not possible at this point to know exactly what the benefits of this dual-action rehydration strategy are to performance. However, based on what is known about the adverse effects of dehydration on performance, and the benefits to better hydration, it is reasonable to predict that performance benefit will follow. The weight at point A in the Control arm (88.59 kg) is effectively the baseline weight prior to training and without any RS intervention. If this weight is used to calculate the change from baseline to the final weight (point C) in each arm, we obtain a complete estimate of benefit of the rehydration strategy to weight. As can be seen from Tables 2, 0.80 kg was lost in Control arm compared to 0.12 kg in the Intervention arm, a difference of 0.68 kg corresponding to an 85% lower reduction in weight. As a percentage of mean body weight the Control arm lost 0.92% (0.80/87.22) compared with 0.13% (0.12/87.22) in the Intervention arm. Given that fluid deficits of 1.65% and above lead to physical and cognitive impairment, this weight benefit may have a positive impact on athlete performance.

## Conclusion

This pragmatic study shows the SpORS hydration strategy to be effective in the sports context. These results reflect better hydration before training and at the completion of training as well as 30–60 min after commencement of the post-training recovery period. This appears to be the first time that a hydration strategy has been shown to result in better hydration prior to exertion. The magnitude of the benefit may be sufficient to improve performance and further studies to test this are now indicated. In view of its demonstrated usefulness in the medical sphere in conditions where dehydration is even more marked, plus the safety and tolerability of the intervention, it is now appropriate to objectively test this strategy in athletes for the purposes of improving performance.

## Abbreviations

ACTRN: Australian New Zealand Clinical Trials Registry
AFL: Australian Football League
GPS: Global Positioning System
HAMS: High Amylose Maize Starch
RS: Resistant Starch
SCFA: Short Chain Fatty Acid
SpORS: Sports Oral Rehydration Solution
UNICEF: United Nations Children’s Fund
WHO: World Health Organisation
